# Acknowledgement to reviewers

**DOI:** 10.1530/ERC-33-A1

**Published:** 2026-01-21

The editorial board wishes to thank the following individuals who have helped to review manuscripts for *Endocrine-Related Cancer* during 2025. Their assistance is gratefully acknowledged.

**Table:** 

E Aalbersberg
N K Aaronson
M Abubakar
A Adamska
D Africander
P Agarwal
R R Aggarwal
S Aggarwal
S Agosto
P Agrawal
D A Ail
T Åkerström
S A Akker
G Akkuş
M Al-Alwan
M Albuja-Cruz
C Aldecoa
K I Alexandraki
A S Ali
O Q B Allela
D B Allison
C E Almasi
J P Almeida
M Almeida
M Q Almeida
M Almquist
T Alonso-Gordoa
P Alonso-Magdalena
T Al-Toubah
C L Alves
A S Alzahrani
R Ambrosini
V Ambrosini
V Amoroso
M Amrutkar
B Anawalt
D Anaya Saenz
L Anderson
C L Andoniadou
M Andreassen
T E Angell
L Annaratone
J Annes
L Antony
L Apostolidis
M Appetecchia
S L April-Monn
M Araujo-Castro
P Arens
M Armanios
E Armeni
M Arnedos
A Arnold
E K Arthur
S L Asa
G Assié
I Attili
E Audet-Walsh
P Auloge
J Austin
I Azaryan
A Azhdarinia
A Azmi
D Bacha
S Backman
C Badiu
J H Baek
R M Baertschiger
M F Baietti
E Baiyee Toegel
D Bajic
S Baldari
D Ball
I Bancos
D Bao
R Bareja
A Barlier
M J Barros
D K Bartsch
J Baselga
K Basham
G A Bass
S Basu
A Bauer
J-P Bayley
M Beato
N Bechmann
A Beckers
D G Behm
A M Bellizzi
D E Benn
A Benrick
A Bereimipour
A M A Berends
E K Bergsland
S Berhane
M C Berkovic
T Berman
A Berruti
L Bertolaccini
F L Besson
C L Bevan
R Beydoun
M Bhandare
N A Bhowmick
C Bi
P Bi
B Biagetti
A C Bianco
B Bianco
R Bianco
T Bianco-Miotto
J Bibb
J A Bibb
L Bischoff
T Bishop
A T Blank
L Bodei
K Boelaert
P Bogdan
A Bojko
J Bollerslev
A Bongiovanni
M Bongiovanni
G Bonomo
M Borahay
G F Borges
K S Borges
V Borges
K Borka
M Bortoli
L Boucai
P Boudou
P Bower
D W Bowles
E Boyle
T Brabander
M Brait Rodrigues de Oliveira
T Brandenburg
H M Brechbuhl
J Brito
K Britt
G Brock
D T Broome
M Brose
M S Brose
S Broutin
A M Brufsky
A Bruni-Cardoso
M Buchfelder
L O Buchmann
A Buffet
C Buffet
M J Bugalho
E Buitenwerf
M Bujko
C J Burd
S A Burgos
N Burnichon
L M Butler
H Butz
X Buy
M E Cabanillas
M L Calcagni
C E Caldon
I Califano
D Campana
K L Campbell
M Campbell
I Campi
G Cantini
G L Canu
L Canu
J Capdevila
B C Capell
V Cappelletti
G Capurso
T Carling
S Carreira
J S Carroll
A Cascón
F Castinetti
R Castro
A Cazes
R A Ceausu
F Ceccato
J M Cerutti
F Cetani
C Chaccour
J A Chan
M D D Chan
E Chang
Y-C Chang
P Chanson
D Chatterjee
N Chatterjee
A Chauhan
Y Che
A M H Chen
D Chen
H Chen
J-H Chen
W Chen
X Cheng
P Chieffi
P Chittiboina
M-H Choi
C N Chougnet
E Christ
D Chung
R Ciampi
S Ciernikova
D M Ciobanu
M Cipolletti
Y Ciribilli
A Cistaro
A Ciuche
A C Civelek
M Cives
F Claessens
R Clarke
H Clevers
R J Clifton-Bligh
D L Cohen
G Colleluori
M P Colombo
B Conte
S Corbetta
F Cordido
F Corti
T Coskun
D A Costa
P Cottu
I Couto-Gonzalez
A Couvelard
R Craver
G A Cremaschi
S F Crino
A Croitoru
J Crona
J Cros
X Cui
E Cukierman
R Cummings
L L Cunha
T Cuny
A Curioni-Fontecedro
E Curtit
A N Cury
A Czerwonka
C Daccord
R Dadu
P L M Dahia
W Dai
F Dalenc
A Dalla Volta
A F Daly
G Dam
R H Dammann
C V Dang
J T Dankert
B Darbro
C Das
G M Das
K Daskalakis
K Datta
L Davies
S R Davis
W W de Herder
E J de Koster
A De la Vieja
S De Leo
M C De Martino
L de Mestier
S M C De Sousa
D Deandreis
T Decker
J Decock
A Degasperi
S M Dehm
J Deinum
J Del Rivero
M J Demeure
M Dentice
K M Devaraj
A Di Cristofano
G Di Dalmazi
N Diano
C Dierks
J J Díez
J S Dillon
G K Dimitriadis
C Dinauer
X-S Ding
M Diniz da Costa
N Ditsch
O Dohan
J Doll
R Dong
S Donzelli
S D'Oronzo
H Dralle
D Drui
M Du
Y Du
M Duan
I Duarte
Q-Y Duh
E Duncan
A Dupré
C Durante
S Dutta
J R Eads
P H Ear
E K Egger
F Eilsberger
R Eisenman
J M Eizenga
I Elfaki
R Elisei
A El-Menyar
T Else
N K Emami
M Endo
F E Escorcia
S Ezzat
A S C Fabricio
A Faggiano
M Falconi
Y Fan
W Faquin
M Fassnacht
J Favier
N Fazio
P Fedele
R Feelders
R A Feelders
W Fendler
F L Fernandes-Rosa
D Ferone
I Figueira
S Filetti
A Fingeret
J Finlay-Schultz
L Fishbein
R Flavell
M Fleseriu
C E Fletcher
J Fletcher
S M J Fliedner
G Fontemaggi
P Formisano
I M Forte
E Fortunati
B A Foster
W Foulkes
C A d C Fraga
M C Fragoso
M Franchi
G Francis
F Frattini
R Frazzi
M Freedman
J French
J A Freyre-Gonzalez
R Fu
L Fugazzola
K Fujita
S Fukushige
E Furth
M Furuya
C T Fuss
F Gabalec
M Gadji
R F Gagel
T Gagliano
A K Gaharwar
M D Gahete
N Galiano-Castillo
A Galimzhanov
F Galuppini
A Gangi
I Ganly
K Garcez
R García-Carbonero
M Gariboldi
D Garrido
V Gasperi
M E Gatti-Mays
S Gaujoux
E Geer
J Geliebter
D Generali
M George
L Gerard
O G Gevaert
H K Ghayee
P M Ghosh
R A Ghossein
L Giacomelli
M Gild
A Gill
O Gimm
T J Giordano
L Giovanella
A Giustina
P Glinicki
D Glintborg
A Glover
A R Glover
Y Godbert
I M Goemann
R Goldberg
W Goldner
T Golias
C C Goller
J Gómez Tejeda Zañudo
N Gontier
M A Gouda
F Gouin
A Goyal
J Graham
C M Grana
D Granberg
E Grande
E Grassi
F Grawe
A Greco
S Greenberg
A Greystoke
V K Grolmusz
M Grönberg
A B Grossman
C Grötzinger
L Groussin
S Grozinsky-Glasberg
E G Grubbs
B Guan
H Guan
C Gubert
J H Gunter
Q Guo
M Gupta
L Gutierrez
C Ha
D Habauzit
M A Habra
J Hadoux
C Hagemann
G Hager
A W Hahn
S Hahner
T R Halfdanarson
J Hallet
R Halperin
M Hamad
S Hamidi
G D Hammer
X Han
A B Hanker
S Hankinson
C Hansen
C Hantel
H Harada
I Harbuz-Miller
C R Harris
M R Harrison
B Haugen
B R Haugen
X He
C M Heaphy
H V Heemers
M Heinaniemi
K Herrmann
G Hickey
R Hicks
R J Hicks
L Hilakivi-Clarke
T D Hinds Jr
G O Hjortland
V Hlavac
J Ho
K K Y Ho
L N Hoang
A O Hoff
J Hofland
M S Hofman
A N Holding
P Holmager
M Holmberg
L Holmquist Mengelbier
J Holst
K Holzer
J Honegger
T Hong
N Hoogerbrugge
K Horiuchi
C Horowitz
D Hörsch
P Hou
D Hrncic
B Hu
M I Hu
R Hu
F Huang
L Huang
S Huang
T-T Huang
A Hubalewska-Dydejczyk
I T Huhtaniemi
N Hulikal
G Huszty
S Huveneers
C Ibanez
M Ibrahim
T Ibrahim
P Igaz
M Ilmer
A Imperiale
C Isaacs
P C Iyer
T Jaber
A Jacob
M Jacobs
W Jacot
J Jagtap
A Jannin
B Jarzab
S Jasim
R Jaskula-Sztul
R Jayaraj
S H Jee
Y H Jee
G Jenster
S R Jeon
T Jerde
R Jeselsohn
M Jesinghaus
S Jha
Q-h Ji
S-S Jiang
X Jiang
C Jimenez
S Jimenez-Morales
C-H Jin
R Johnson
E Jonasch
N Jonckheere
J Jonklaas
J Jorgensen
N M Joseph
E Jouanneau
A Jouinot
P Joyce
C C Juhlin
S J Jung
S-H Jung
D Kaemmerer
B A Kaipparettu
D J Kalita
M Kalita
G Kaltsas
G A Kaltsas
A F Kamal
S Kanchan
E Kandil
R Kandimalla
F Kang
Y Kang
Y E Kang
R Kapoor
I Karfis
A Kasajima
L Kassem
I Kastrati
S Kaur
S Kaushik
E Kebebew
M Kebede
F Kelestimur
H Kelestimur
Z Kender
H F Kennecke
M N Kerstens
X Keutgen
X M Keutgen
E Khaleel
A A Khalil
M S Khan
B N Kholodenko
T Khoury
B Kiesewetter
D S Kim
J Kim
J H Kim
K J Kim
M Kim
S Kim
S Y Kim
W G Kim
T Kimura
J Kirani Tan
L S Kirschner
K Kiseljak-Vassiliades
C Kitahara
C M Kitahara
J Kitlinska
D A Kleiman
B K Kleinschmidt-DeMasters
D Kletsas
H Klocker
G Klöppel
J Klubo-Gwiezdzinska
J A Knauf
T Knösel
J J Knox
K Koga
T Kogai
M Kohli
Z Koledova
D L Kolin
B Konda
M Korbonits
M Korbonitz
K Korthauer
B Kos-Kudla
S Kotamraju
A Kotwal
N Koufopoulos
H Koul
A Koumarianou
B P Kovari
J Krajewska
P Krawczyk
M Krebs
M C Kreissl
R Kremer
G Kroemer
M Kroiss
H Krude
S Kruijff
M Kruithof de Julio
C R Ku
A Kudo
A Kulasinghe
J Kulka
P Kunz
J Kuo
T R Kurzawinski
K Kusnierz
H Y Kwan
M Kwan
D Kwekkeboom
A La Salvia
N Lack
M Ladurner
T Laetsch
A Laffi
M Laiho
E Lalli
A K-y Lam
A Lamarca
G Lamberti
I Landa
L Landuzzi
L Lanfrancone
J E Lang
J M Lang
T Lange
J F Langenhuijsen
F Langlois
A G A Lania
L Larue
L Latonen
Y Latunde-Dada
M Lauseker
G Laverny
M G Lawrence
S L Lay
I M Lazar
M Le Romancer
D Leahy
C A Lebbink
S Leboulleux
M Lechner
C H Lee
C-H Lee
M Lee
B Lehman
B D Lehmann
K Lehmann
J Lei
J L Leight
M Lemmon
D Leong
A M Lerario
E Leucci
R Leung
Y-k Leung
M A Levine
M Lewis
B Li
C Li
J Li
J-M Li
L Li
M Li
Q Li
S Li
X Li
Z Li
G Li Volti
R Libé
B Lim
K H J Lim
Y Limami
A Lin
H Lin
K E Lines
T P Links
S Liu
X Liu
Y Liu
M J Livhits
A W I Lo
L D Locati
M Lodish
H Loomans-Krop
P Lopez
K Lorenz
A Lorico
C F P Lotfi
C Lottspeich
G Lotz
P B Loughrey
A F Louis-Jacques
J Lu
I Luciene da Silva
M Luconi
K Lueckerath
A Luiz Vettore de Oliveira
S Lupold
R M Luque
C Lussey
M Luster
L Ma
S Ma
X Ma
S Maasberg
J A Machado-Neto
A Machens
Y M Machlah
M Macias-Gonzalez
R M B Maciel
Z Madak-Erdogan
L J Maher
N Mahmud
A Mahran
A L Maia
H J Majima
M S Makary
P Malandrino
A Malayeri
A Mali
L Malorni
F Mangili
A Mangogna
A Maniakas
W Mansoor
M Marazuela
S Marco-Sola
A Marfak
A G Maria
P Marini
D Marino
B A Marks
J Marquez
L Marroqui
D J Marsh
J G Marshall
C S Martin
F Martorana
A Matrone
D Matvienko
G Mauri
L J Mauro
J E Maxwell
R Mazzilli
B Mazzoleni
F Mazzoni
M Mazzoni
C J McCabe
A E McCart Reed
H McDaid
M McIlroy
R McIntyre
K Meehan
R Melillo
I M Meraz
L Mercado-Asis
A Mercurio
L S Meyer
M Meyerson
I Michael
M Michael
T Michalopoulou
P Michl
C T Mierke
D S Mikhaylenko
R Mikolajczak
M Mileva
T W Miller
I M Min
E Minna
S Minner
C Miranti
A L Mitchell
S Mitola
N Mitsiades
R Modica
L C Moeller
M Moh
J Molina-Cerrillo
L Möller
M E Monaco
R Q Monteiro
F Montemurro
B H M Mooers
S Moog
L Morelli
A Moreno-De-Luca
T Moriya
A Motyka-Pomagruk
D Mourtzoukou
M H Muders
M Mudryj
S Mukherjee
C Muller
L M Mulligan
A Munir
A Murad
B D Murphy
L C Murphy
T J Murtola
A Muth
M Muzza
F Nabhan
B Nagy
H Nakagawa
Y Nakamura
S Nandana
A Nappi
B Naraev
M Naruse
C Nasr
G Nassa
K L Nathanson
S J C M M Neggers
R T Netea-Maier
N Nevin
K Newbold
P J Newey
R Newton
J Ngeow
F Nguyen
Y Ni
E Niccolai
J L Nicklin
J Nicola
G P Nicolas
D Niculescu
M B Niederle
P S Nielsen
M Niemira
M Niger
Y E Nikiforov
M N Nikiforova
N Nilubol
S Ning
S Niraula
Y Nishida
T Nishikawa
S Nölting
E Nordenström
O Norlén
A M Novak
C Nucera
P Nuciforo
G Nyirő
K E Oberg
D O'Connor
T O'Dorisio
S Oesterreich
K Ohashi
A Ohmoto
Y Okita
D O'Malley
S Omarjee
T Ones
T Ord
H U Osmanbeyoglu
C O'Sullivan
D M Otterburn
M Oudin
Y Ozaki
K Pacak
R Pacholczak-Madej
R d P Padovani
M Padro Perez
E Palmerini
G Palmini
C Pamporaki
S Panza
F Panzuto
G Papadakis
G Z Papadakis
M Papaleontiou
U-F Pape
M Parasiliti Caprino
P Parente
J Park
S W Park
S-Y Park
Y S Park
C Parolini
A Parrales-Bahena
C Parry
S Partelli
A Pascher
A Passaro
N Patel
M S Paulo
M E Pavel
A Paziewska
A Pearson
R P Peeters
V Peg
N Pellegata
N S Pellegata
G Pellegriti
S Pereira
L G Perez-Rivas
A Perren
M Perrier
N Perrier
L C Perrin
L Pesce
B J Petri
I Petrini
E Peverelli
R Pezzani
J Phay
J Piao
C R C Pieterman
J Pietzsch
R Pimenta
E M Pinto
Z Piperigkou
S Piscuoglio
F Pitoia
S Pittock
C Pivonello
M Pizon
H Plaudis
J K Plichta
J Pohlenz
P Pohlmann
A M Poma
L-A Pop
F L Popa
V Popovic
P Popovics
K G Poppe
C Porta
C Poulard
J F Powers
N Pozdeyev
D Pradhan
V Prasad
N Prinzi
J Przybyl
A Ptak
T Puar
M Puig-Domingo
A Puri
C Pusceddu
S Pusceddu
K Qian Luo
N Qu
D Quelle
A-M Quigley
D Quigley
C Quinn
D Rades
A Rafacho
M Ragni
E Rahrmann
M Raica
W Rainey
N Raj
N Rajan
J K Ramage
R A Ramirez
G Randolph
G W Randolph
P V Raninga
E Rapizzi
F Raue
S Ravaioli
G Raverot
M L Read
I Reccia
D J Reeves
D Reidy-Lagunes
B Rekhi
Y Ren
P C N Rensen
M Reyazi
O Reyes
C Ricciardelli
S Richter
J Ridge
J A Ridge
F Ridouani
R P Riechelmann
M Riedmeier
R B Riggins
M D Ringel
A Rinke
R Robertis
N Robine
M Robledo
V Rochira
C Roderburg
P Rodien
V Rodilla
M Romero-Arenas
C L Ronchi
P W Rosario
J B Rose
J Roy
S S Roy
S M Ruff
R M Ruggeri
G Ruli
N Russell
C S Rutland
P Ruusuvuori
M Ryder
P A Saa
S F Sabet
P M Sadow
V A Saenko
A Saiga
G Salvatore
M Sandstrom
G Sanghvi
A Santos
M Santos
V Santosh
G Sapuppo
M Saracyn
A Sarma
M Sarquis
K Sasaki
A Sasson
A Sato
E C Sattler
S Sawicki
G Schembri
A Schieber
M Schlumberger
J J Schmitz
C Schneider
J Schrader
R Schulz
R E Schweppe
T Seagroves
F Secchi
M Sedrak
B Seeliger
J Segars
V R Seifert-Klauss
A Selberherr
L A Selth
A H Shah
S Shaheen
A Shajahan-Haq
O Shariq
A Sharma
B M Sharp
C Shen
M Shen
S Shepherd
B Shi
Y Shi
M Shibata
I Shin
M Shiota
R Shiradkar
S Siddharth
S B Sidhu
I Sidorkiewicz
S Sigala
W F Simonds
A Simon-Soro
S Singh
S Singhal
D Sinha
L Sinkkonen
D Sisci
M Sjostrom
A Skalniak
A Skardal
J Smith
P W Smith
V E Smith
P Soares
B Soldevilla
S Soliman
A Somma
M H Son
S Sona
E J Song
G Song
H Sorbye
K Soreide
I C Sormaz
J A Sosa
K Souček
M C Soulen
C Soydal
F Spada
N Spakowicz
P N Span
H Speckter
D Spiegelberg
C Spitzweg
M Sprick
R Srirajaskanthan
M N Stan
S Stattner
M Steensma
M Stefanick
M Stellato
H A Sterle
D Steward
D R Stewart
C A Stratakis
J R Strosberg
M M Stuiver
V Subbiah
K Sugano
V Sukrithan
P E Summers
Y Summers
W Sun
Y Sun
A Sundin
A Sundlöv
L Suva
V Swaminathan
S Swaroop
M Sywak
M Tabebi
M Tacelli
E Tagliabue
D Taieb
M Takahashi
A Takeuchi
A Tanabe
P K Tandra
M Tang
C Tatsi
V Teleanu
T Terashima
M Terzolo
M Tetti
A Teule Vega
P S Thakuri
M Theodoropoulou
C Thirlwell
C K Thomas
R Thomas
C Thomssen
G J Thorn
Z Tian
G A M Tiberio
V Tiedje
E Tiensuu Janson
W D Tilley
H J L M Timmers
A Tirosh
A Tischler
A S Tischler
M Toi
J Toke
R Toledo
A Tong
L Torregrossa
F Torresan
E Toska
M Tóth
R W Tothill
L M Trandafir
D Trapani
G Treglia
J Trent
P Trimboli
F Triponez
G Trivellin
T H Truong
K Tsilidis
M Tsoli
E Tsourdi
R Tufano
R P Tufano
G Tuli
J Tumas
S Turajlic
C G Turin
R M Tuttle
S Uccella
S Uchino
T Ueno
C Ugolini
A Uhlyarik
R N Uppot
A Urbschat
P Uysal-Onganer
V Vafeiadou
F Vaisman
P Val
N Valdes
N Valdés
J Valle
J W Valle
M F M van den Broek
G van der Pluijm
R S van Leeuwaarde
D Van Nostrand
M J C van Treijen
M-L F Van Velthuysen
T Vandamme
L N Vandenberg
D J Vander Griend
S Vasudevan
S C Vaz
V G Vellone
G Venkatraman
C Vernieri
G Viale
G Vila
V E Villegas
G Vitale
M Volante
D Vordermark
O Vycital
H Wachtel
S G Waguespack
J Waisberg
A M E Walenkamp
M Wallace
T Walter
D Wang
H Wang
R Wang
T Wang
Y Wang
L S Ward
J D Wasserman
A Wassner
B H Wattar
F Weber
M Weber
E Wedi
R Wei
V Weiss
F Weitzer
U Wellner
V U J Wenter
B Whitelaw
F Wiede
V Wiegering
H Wikman
D Wild
C Williams
T Williams
L Wirth
L J Wirth
I Witzel
R M F Wolthuis
R J Wong
F Worden
F P Worden
T Worst
P K Wright
B J Wu
H Wu
J Wu
Z Wu
F Wu
H Xiang
W Xie
M Xing
C Xirouchaki
Z Xu
M Yadav
S K Yadav
J W P Yam
A Yamamoto
Y Yamazaki
J Yan
R Yan
D Yang
H Yang
J Yang
Y Yang
Z Yang
J Yao
J C Yao
Y S Yap
K Yashima
H Yasuda
M Ye
L Yehia
G Yeo
H C H Yim
D-t Yin
J Y Yoo
A E Yu
H Yu
S-T Yu
W Yu
X Yu
M Zacherl
M Zafereo
V Zaia
W Zandee
L Zanoni
M Zatelli
M C Zatelli
F Zenke
M C N Zerbini
W Zeyghami
H Zhang
J V Zhang
Q Zhang
W Zhang
Y Zhang
G Zhao
P Zhao
Y Zhao
X Zheng
Z Zheng
J Zhou
Z Zhou
Z-Y Zhou
D Zhu
L Zhu
Y Zhu
A Zielke
A Zimpfer
X Zong
E Zoni
A Zoubeidi
W Zwart

